# Talquetamab induces deep responses in heavily pre-treated patients with systemic light-chain amyloidosis

**DOI:** 10.1007/s00277-026-06931-3

**Published:** 2026-03-12

**Authors:** Tamir Shragai, Muhammad Ganayem, Yael C. Cohen, Irit Avivi, Eyal Lebel, Noa Even-Gross Zohar, Natan Melamed, Moshe E. Gatt

**Affiliations:** 1 Department of Hematology, Tel-Aviv Sourasky Medical Center, Tel-Aviv, Israel; 2 Gray Faculty of Medicine, Tel-Aviv University, Tel-Aviv, Israel; 3 Department of Hematology, Hadassah Medical Center, Jerusalem, Israel; 4 Hadassah Medical School, Hebrew University, Jerusalem, Israel

**Keywords:** Light-chain amyloidosis, Relapse/refractory amyloidosis, T-cell engagers, Plasma cell disorders

## Abstract

Talquetamab, a CD3/GPRC5D T-cell engager approved for triple class exposed myeloma patients, inducing deep and durable responses. Very few cases of relapsed/refractory (R/R) light chain (AL) amyloidosis patients treated with talquetamab, were reported to date. We report six heavily pretreated R/R AL amyloidosis patients with severe end-organ damage (five with cardiac involvement), treated with talquetamab. Talquetamab induced rapid and deep responses: Five patients achieved complete response. (including MRD negativity in all three evaluable patients). At data cut-off, three patients were alive and relapse-free at 8, 15 and 24 months, and three patients died, after 1, 1.5 and 5 months since initiation of treatment. Five patients were not evaluable for organ response: (Three due to end-organ kidney disease, and two who died before organ response assessment). One patient achieved cardiac response. Two patients were referred to kidney transplantation after achieving CR. Cytokine release syndrome occurred in four patients, all grade 1–2, and immune effector cell associated neurotoxicity syndrome (ICANS) was reported in one patient (grade 2). Infections occurred in two patients (one grade 3, one grade 5). Congestive heart failure exacerbation occurred in two patients (grades 3 and 4). Our results support talquetamab as an effective therapy for RRAL patients, although larger-scale studies are needed to optimize earlier timing and patient selection.

## Background

 Light-chain (AL) amyloidosis is a rare plasma cell dyscrasia caused by unstable misfolded light chains, produced by plasma cell clone, which is prone to form amyloid fibrils that infiltrate end-organs and cause devastating organ damage [1]. Achieving deep responses is crucial for enabling target organ responses. Treatment is based on suppression of the light-chain secreting plasma cells using multiple myeloma (MM) directed regimens. Therapy with monoclonal antibody to CD38, proteosome inhibitor, cyclophosphamide and dexamethasone are the standard of care frontline therapy [2], resulting in high rates of hematological and organ responses. Treatment alternatives are scarce for patients who relapse following Daratumumab- based regimens.Patients with t(11;14) have favorable response to the BCL2 inhibitor venetoclax [3]. Recent evidence supports the efficacy of B-cell maturation antigen (BCMA)-targeted agents, including chimeric antigen receptor T-cell therapy (CAR-T) [4], antibody-drug conjugates [5–7] and T-cell engagers (TCEs) [8–11]. Talquetamab is a T-cell engager targeting G protein–coupled receptor class C group 5 member D (GPRC5D) with proven efficacy in heavily pretreated MM patients, but also with on-target off tumor toxicity, mainly dysgeusia and weight loss. To date, only 5 relapse/refractory (R/R) AL amyloidosis patients treated with talquetamab were reported, in 2 different reports [11, 12]. Presented here is a six-patient case series comprising five new patients and extended follow-up of the index case previously reported.

## Methods

This is a retrospective case-series, including all adult patients with biopsy-proven RR AL amyloidosis and treated with talquetamab in two centers. The data cutoff for the analysis was Jan 1st, 2026. This study was reviewed and approved by local institutional review boards of Tel-Aviv sourasky medical center (study number: TLV-18-0362) and Hadassah medical center (study number: HMO-11-06920), and conducted in accordance with the Declaration of Helsinki. All patients provided written informed consent for data collection. All patients received step‐up dosing of talquetamab according to the prescribing label information. Data was extracted from medical electronic charts focusing on demographics, AL-involved organs, related biomarkers, previous treatment regimens and the presence of active MM defined by the presence of one or more myeloma defining events, namely AL/MM [13]. The revised Mayo Clinic Criteria were used to stage cardiac amyloidosis [14]. The hematologic and organ responses were assessed as previously described [1, 15, 16]. Minimal residual disease (MRD) was assessed using fluorescence activated cell sorting at a sensitivity threshold of 10^− 5^. Adverse events (AEs) were graded based on the common terminology criteria for AE (CTCAE) (https://dctd.cancer.gov/research/ctep-trials/for-sites/adverse-events/ctcae-v5-5x7.pdf). Cytokine release syndrome (CRS) and immune effector cells associated syndrome (ICANS) was graded according to the ASTCT consensus scale [17]. Progression-free survival (PFS) was defined as the time from 1st talquetamab dose to progression, death or last date of follow-up, and overall survival (OS) was defined as the time from initiation of talquetamab until death or last date of follow-up.

## Results

Six patients (five males), median age 63 years (range 53–74), were included. Two had concurrent MM. Heart and kidneys were the most commonly involved organs (five patients each). Other systemic involvement included peripheral nervous system and soft tissue (3 patients each), gastro-intestinal (2 patients) and liver involvement in one patient. Three patients had ≥ 3 organs involved at baseline (Table 1).

**Table 1:** Patient characteristics

patient no.	Age at diagnosis	Sex	BM PC (%)	Involved light chain (Kappa/Lambda)	FISH	Involved organs	Revised Mayo staging (1-4)	Concurrent MM	Previous therapies
1	45	F	10	K	4:14	Heart, Kidney, Liver, PNS, Soft tissue, GI	N/A	No	V, D, C, P, Len, B, ASCT (X2), CART, LDM
2	68	M	60	K	del 13q l	Heart, Kidney	2	Yes	V, D, Len, P, B, ASCT
3	53	M	10	K	del13q 1q gain IGH rearrangement	Kidney	2	No	V, D, C, P,
4	67	M	15	K	Normal	Heart, Kidney	4	No	V, D, C, P,
5	56	M	60	L	1q gain, del 17p	Heart, Kidney, PNS, Soft tissue	2	Yes	V, D, C, P, Len, B, ASCT, CART, K, I
6	57	M	15	L	11:14	Heart, PNS, Soft tissue, GI	3	No	V, D, C, P, Len, B, ASCT, Ven, E, I, CART
patient no.	Reason for TAL administration		Age at TAL initiation	dFLC at TAL initiation	eGFR (MDRD) at TAL initiation	Dialysis dependent	NT- PROBNP^#^ prior to TAL	TAL dosing	
1	PD		60	91	50	No	570	0.8 mg/kg every two weeks	
2	insufficient response		74	8	15	Yes	N/A	0.8 mg/kg every two weeks	
3	PD		53	219	11	No	N/A	0.8 mg/kg every two weeks	
4	insufficient response		69	4	12	Yes	N/A	0.4 mg/kg weekly	
5	PD		67	410	28	No	4287	0.4 mg/kg weekly	
6	PD		63	383	41	No	9,720	0.4 mg/kg weekly	

F – Female, M – Male, K – Kappa, L – Lambda, V – Bortezomib, D – Daratumumab, C – Cyclophosphamide, P – Pomalidomide, Len – Lenalidomide, B – Belantamab, ASCT – Autologous Stem Cell Transplantation, CART - Chimeric Antigen Receptor T-cell, LDM – Low dose melphalan, K – Carfilzomib, I – Ixazomib, Ven – Venetoclax, E – Elotuzumab, PD – Progressive disease, MM- multiple myeloma, TAL: talquetamab, dFLC: involved light- uninvolved light chain, eGFR- estimated glomerular filtration rate, NT PROBNP: N-terminal pro-B-type natriuretic peptide, N/A – Not applicable

^#^NT- PROBNP measurement of patients with end stage kidney disease were not recorded

At talquetamab initiation, two patients had Mayo stage 3 or 4 disease. All had renal impairment (eGFR 11–50 mL/min/1.73 m²), including two dialysis-dependent patients; NT-proBNP (applicable in patients without end-stage renal disease [ESRD]) was 570, 4,287, and 9,720 pg/mL. Abnormal cytogenetics were present in five patients, most commonly 1q gain (two; one with del17p) and del13 (two) and a single cases of t(4;14) and t(11;14) each. Patients had received a median of 7 prior lines (range 2–11) and were universally refractory to daratumumab, bortezomib, cyclophosphamide, and pomalidomide; four were also refractory to lenalidomide and belantamab mafodotin, and one patient with t(11;14) patient was refractory to venetoclax. Three patients relapsed after academic anti-BCMA CAR-T (HBI0101). Talquetamab was started for progressive disease in four patients and inadequate response in two, in a median of 71 months (range 8–132) from diagnosis and was administered for a median of 5.2 cycles (range 1–22) at 0.8 mg/kg every other week (four) or 0.4 mg/kg weekly (two).

### Safety

**Fig. 1:**
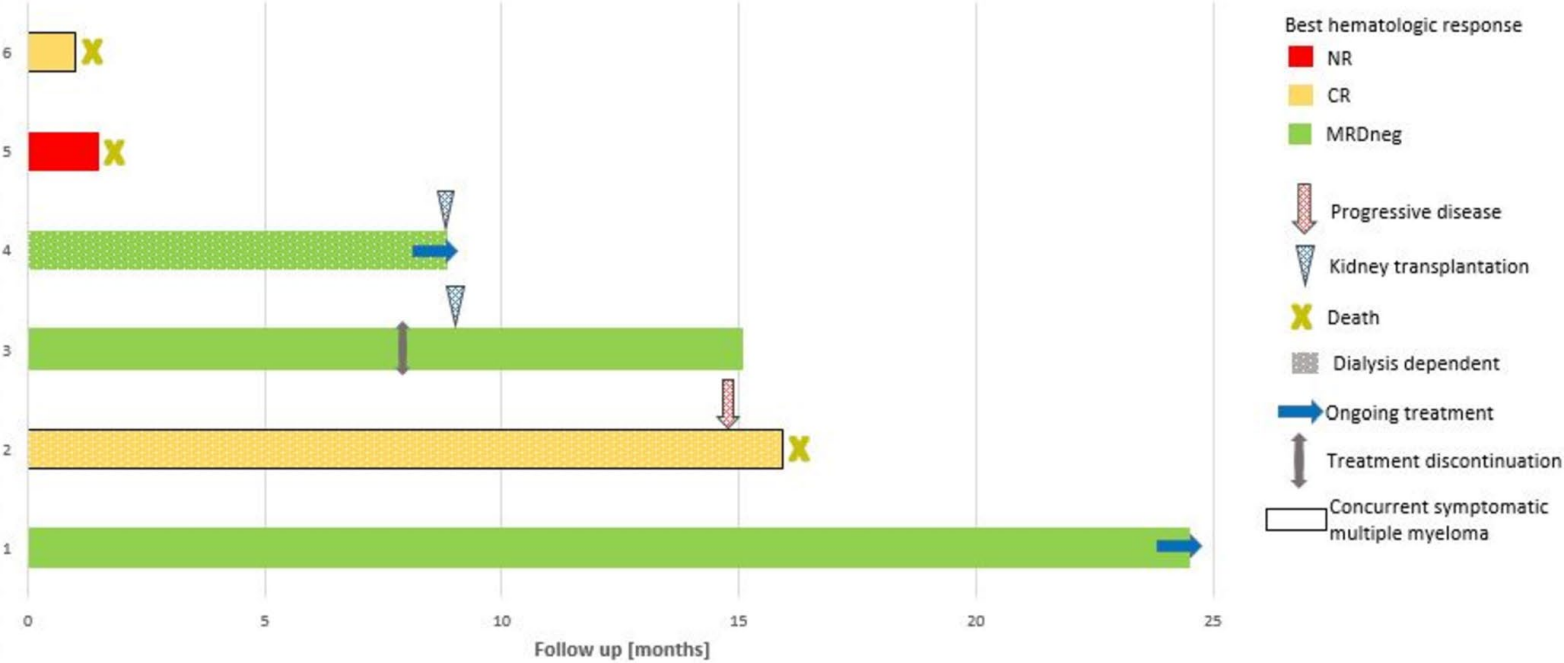
Longitudunal follow-up of relapse/refractory AL patients treated with talquetamab

CRS occurred in four patients (all grade 1–2) and ICANS in one (grade 2). Severe infections occurred in two patients (one grade 3, one grade 5) and transient exacerbation of congestive heart failure in two (grades 3 and 4). Additional adverse events included: dysgeusia (grade 2), xerostomia (grade 2) and weight loss (grade 2) in two patients each; anemia (grade 3), fever and thrombocytopenia (grade 2) in one patient each. Three patients died: One relapsed five months after talquetamab initiation and died from sepsis, one died from cardiac failure despite achieving CR, and one who was primary refractory (Fig. 1). None of these were attributed to talquetamab. Time from talquetamab initiation to death was five months, six weeks and four weeks, respectively.

### Efficacy

**Fig. 2:**
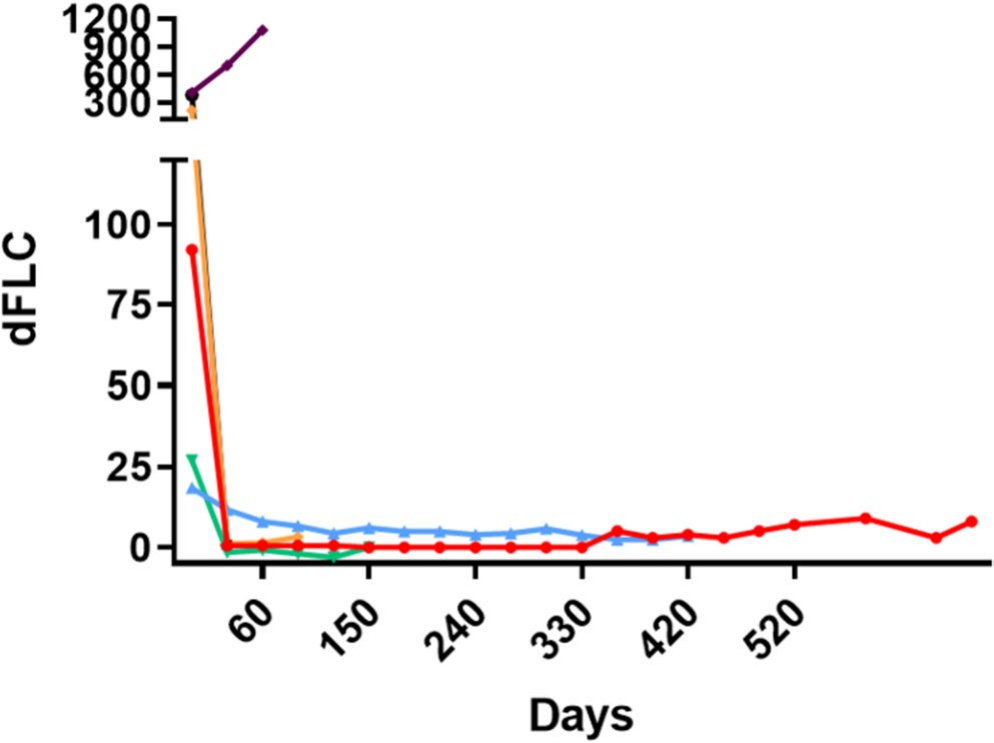
Free-light chains dynamics post treatment with talquetmab. dFLC: involved light- uninvolved light chain

Five patients (83%) achieved a hematologic response, all attaining complete remission (Fig. 2). MRD was negative in 3/3 patients tested, including two patients with sustained MRD negativity (after six months for one patient and nine for the other); the third patient was not re-tested for MRD. The median time to documented hematological response was 26 (range 21–63) days. The median follow-up time was 9.6 (range 1–22) months. At data cutoff, three patients were alive (all in CR) after 24, 15 and 9 months from talquetamab first dose. None of the patients received subsequent therapy. Cardiac response was assessed in two patients: one achieved complete cardiac response, while the primary refractory patient showed cardiac progression. None of the patients were eligible for renal response assessment: two had ESRD that required renal replacement therapy with dialysis, two due to early death and one had insufficient data. Two patients with ESRD were referred to kidney transplantation (KT): One underwent KT nine months after talquetamab initiation successfully. At the time of data cut-off, he is seven months off-treatment and remained in CR (MRD status was not re-evaluated). The second patient underwent KT one week before the data cut-off.

## Discussion

AL amyloidosis patients who progressed after DARA based regimens (and after venetoclax-based therapy for patients with t(11;14)) have very poor prognosis [18]. Data on T-cell redirecting therapy in AL amyloidosis are encouraging, yet limited. Small retrospective series of BCMA-directed TCE treated patients report high hematologic response rates (≥ VGPR in 73–88%) and emerging organ responses [8–11]. No high-grade CRS were reported, and only one case of non-fatal grade 3 ICANS. Mortality rates were low, mostly from sepsis and cardiac death (in patients with advanced cardiac failure prior to TCE initiation). Five patients treated with talquetamab were reported thus far in the literature; Rees et al. [11] reported four patients treated with talquetamab as a part of the largest report on TCE in AL amyloidosis, but the authors did not report efficacy and non-fatal AEs according to the specific TCE given. One patient in their cohort treated with talquetamab died from sepsis two months after talquetamab initiation. Even-Zohar et al. [12] reported the first patient treated with talquetamab for AL amyloidosis, and his extended follow-up is included herein.

The patients in our series were very heavily pretreated, more than previously described for other TCEs, with a median of 7 prior lines, and most of them were virtually refractory to all approved treatments for AL amyloidosis. Most patients had additional significant comorbidities, such as advanced renal failure (including two patients who were dialysis dependent and another one with stage 3B renal failure who were referred to kidney transplantation) and three patients with severe CHF. Talquetamab seemed to induce deep response (5/6 patients achieved CR, and 3/3 tested achieved MRD negativity). The rapid, deep hematologic responses translated into meaningful clinical benefit: one patient remains in remission at 24 months, and two patients with ESRD underwent kidney transplantation. One of them is currently six months post transplantation and remains in CR with good graft function, despite stopping talquetamab seven months prior to last follow-up.

Early toxicity (CRS and ICANS) rates were consistent with clinical trials of talquetamab for RRMM (despite the inclusion of patients with significant heart and renal failure), with all events being grade 2 or less. Congestive heart failure exacerbations are consistent with the clinical course of cardiac AL amyloidosis and may occur with many therapies used in this setting. In addition, all patients received dexamethasone at substantial doses as premedication, which may have contributed. Two patients died soon after talquetamab initiation (four and six weeks), both from heart failure; One of them was refractory to talquetamab and the second achieved hematologic CR but already had severe heart failure (with NT-PROBNP 9720 pg/l) at study entry. His death most likely represents the natural history of advanced cardiac AL amyloidosis patients. This early cardiac death was not attributed to talquetamab, but rather to persistent, end-stage cardiac involvement with irreversible organ damage. While prior multiple myeloma trials [19, 20] did not report significant cardiac toxicity, GPRC5D expression has been described in cardiac tissue [21], and the adverse events observed here may be more clinically consequential in frail patients with cardiac AL. Accordingly, talquetamab should be used with caution in patients with cardiac AL amyloidosis, and its use earlier in the disease course may warrant consideration.

To conclude, in this small case series of heavily pre-treated AL amyloidosis patients with advanced end-organ damage, talquetamab induced deep hematologic responses and meaningful clinical benefit with manageable toxicity and no new safety signals, although caution is warranted in frail patients with severe cardiac involvement. Future prospective clinical trials are needed to better define its safety, optimal timing, and efficacy in AL amyloidosis, particularly in patients with cardiac disease.

## Data Availability

The data that support the findings of this study are not openly available due to reasons of sensitivity and are available from the corresponding author upon reasonable request.
